# Supplementary material for: On the Precipice of Extinction: Genetic Data in the Conservation Management of In Situ and Ex Situ Collections of the Critically Endangered Muehlenbeckia tuggeranong (Tuggeranong Lignum)
Source: Plants (Basel). 2025 Jun 12;14(12):1812. doi: 10.3390/plants14121812 (PMC12197067; doi:10.3390/plants14121812)
Supplement: Supplementary file 1 [file plants-14-01812-s001.zip › plants-3629541-supplementary.pdf]

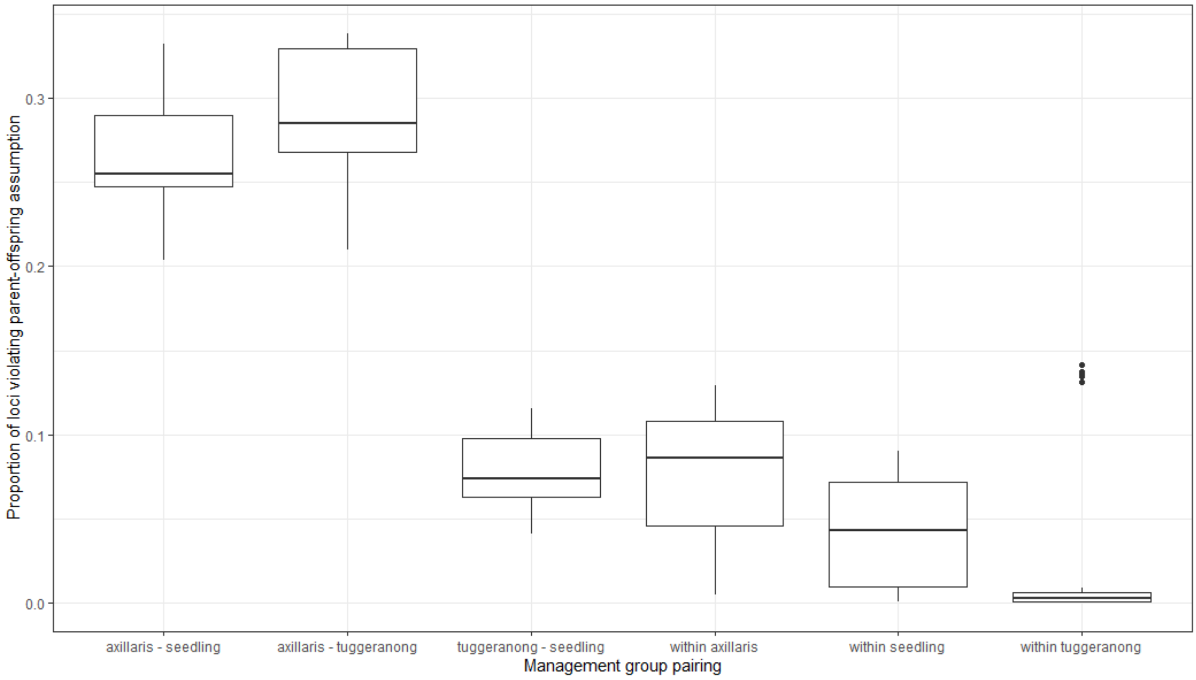


**Figure S1**: Proportion of loci that violate parent-offspring assumption between M. tuggeranong and M. axillaris management groups.
